# Supplementary material for: HIVprotI: an integrated web based platform for prediction and design of HIV proteins inhibitors
Source: J Cheminform. 2018 Mar 9;10:12. doi: 10.1186/s13321-018-0266-y (PMC5845081; doi:10.1186/s13321-018-0266-y)
Supplement: Supplementary file 2 — Additional file 2. Source code of HIVProtI web server. [file 13321_2018_266_MOESM2_ESM.zip › HIVprotI_Source-code/hivprotI/ic50_submit_draw.php]

HIVprotI


php include("head.php");
$ran = $\_REQUEST['ran'];
$ic=$\_REQUEST['ic'];
#$email=$\_REQUEST['email'];
$dir="/home/gpsr/webserver/cgidocs/tmp/manojk/hivproti/first\_$ran";
?

if (file\_exists("$dir/out\_tsort"))
{
echo"
<br  
  
  

**Predicted IC_50_ of query structure on the selected HIV proteins.**
  
  
  
";
exec("perl -pi -e 's/INT/Integrase/g' $dir/out\_tsort");
exec("perl -pi -e 's/PR/Protease/g' $dir/out\_tsort");
exec("perl -pi -e 's/RT/Reverse transcriptase/g' $dir/out\_tsort");
$sort=file("$dir/out\_tsort");
ECHO"

|  |  |  |  |  |  |  |  |  |
| --- | --- | --- | --- | --- | --- | --- | --- | --- |
| "; include("ic50\_topkey1"); $i=0; foreach ($sort as $line){$i++; $cell=explode("\t",$line); if($cell[1] < $ic){ echo "\|  \|  \|  \| \| --- \| --- \| --- \| \| **$i** \| **$cell[0]** \| **$cell[1]** \| ";} elseif($cell[1] > $ic){ $cell[1]=$cell[1]/1000; $cell[1]=round ($cell[1],2); echo "\| **$i** \| **$cell[0]** \| **$cell[1]** \| ";} $FP1=fopen("$dir/graph\_$i","w"); $r=$cell[0];$k=$cell[1]; fwrite($FP1,"['$r', $k]"); fclose($FP1); } exec("cat $dir/graph\_\* >$dir/graph\_total"); include("bottom1"); $x=file\_get\_contents("$dir/graph\_total"); $x=str\_replace("][","], [",$x); $xx=str\_replace("], [","]\n[",$x); $xxx=str\_replace("]"," ",$xx); $xxxx=str\_replace("["," ",$xxx); ##### Download ######### #print""; #echo""; #echo""; #echo""; #echo""; #print" "; #echo "  HIVProtI Source Code       **Download**  "; $FP6=fopen("$dir/ic50\_predict.txt","w"); fwrite($FP6,"$xxxx"); fclose($FP6); echo " "; #**Download**  "; ####################### $FP2=fopen("$dir/graph\_total1","w"); fwrite($FP2,"$x"); fclose($FP2); ECHO" |  | "; echo " |

";
echo"  
  
  
  
  
";
########Plot Graph#########
/\*echo "


";\*/
/\*
##### email sending####
$to = $email;
$subject = 'AVCpred results';
$bound\_text = "AVCpred results";
$bound = "--".$bound\_text."\r\n";
$bound\_last = "--".$bound\_text."--\r\n";
$headers = "From: manojk@imtech.res.in \r\n";
$headers .= "Reply-To: manojk@imtech.res.in \r\n";
$headers .= "MIME-Version: 1.0\r\n"
."Content-Type: multipart/mixed; boundary=\"$bound\_text\"";
$message .= "If you can see this MIME than your client doesn't accept MIME types!\r\n"
.$bound;
$message .= "Content-Type: text/html; charset=\"iso-8859-1\"\r\n"
."Content-Transfer-Encoding: 7bit\r\n\r\n"
."Dear User\n  
 Thanks for using AVCpred. Result of your query is available from URL http://crdd.osdd.net/servers/avcpred/submit\_draw.php?ran=$ran&email=$email , your result will be available for next five days.  
  
 \n\r With Regards \n\r   
  
Manoj Kumar \n\r   
 \n\r";
if($email!=""){mail($to, $subject, $message, $headers);}
#######################\*/
}
elseif($email==""){
echo"  
  
  
  
  
  
";
echo "

#### Please wait....

";
echo "";}
else{
echo"  
  
  
  
  
  
";
echo "

#### Please wait....

";
echo "";}
?>
php include "foot.php"; ?
